# Redox Imbalance as a Common Pathogenic Factor Linking Hearing Loss and Cognitive Decline

**DOI:** 10.3390/antiox12020332

**Published:** 2023-01-31

**Authors:** Fabiola Paciello, Cristian Ripoli, Anna Rita Fetoni, Claudio Grassi

**Affiliations:** 1 Department of Neuroscience, Università Cattolica del Sacro Cuore, 00168 Rome, Italy; 2 Fondazione Policlinico Universitario A. Gemelli IRCCS, 00168 Rome, Italy; 3 Unit of Audiology, Department of Neuroscience, Università degli Studi di Napoli Federico II, 80138 Naples, Italy

**Keywords:** hearing loss, neurodegenerative disease, oxidative stress, antioxidant therapy

## Abstract

Experimental and clinical data suggest a tight link between hearing and cognitive functions under both physiological and pathological conditions. Indeed, hearing perception requires high-level cognitive processes, and its alterations have been considered a risk factor for cognitive decline. Thus, identifying common pathogenic determinants of hearing loss and neurodegenerative disease is challenging. Here, we focused on redox status imbalance as a possible common pathological mechanism linking hearing and cognitive dysfunctions. Oxidative stress plays a critical role in cochlear damage occurring during aging, as well as in that induced by exogenous factors, including noise. At the same time, increased oxidative stress in medio-temporal brain regions, including the hippocampus, is a hallmark of neurodegenerative disorders like Alzheimer’s disease. As such, antioxidant therapy seems to be a promising approach to prevent and/or counteract both sensory and cognitive neurodegeneration. Here, we review experimental evidence suggesting that redox imbalance is a key pathogenetic factor underlying the association between sensorineural hearing loss and neurodegenerative diseases. A greater understanding of the pathophysiological mechanisms shared by these two diseased conditions will hopefully provide relevant information to develop innovative and effective therapeutic strategies.

## 1. Introduction

Numerous clinical and epidemiological studies have highlighted a tight association between hearing loss and cognitive decline [1,2,3,4]. Accordingly, a significant prevalence of cognitive dysfunctions has been observed in presbycusis patients [5]. Clinical studies reported that subjects with mild or moderate presbycusis show an impairment in memory and executive functions [6] and that hearing loss is correlated to global cognitive decline, both factors synergistically contributing to social isolation and depression [7]. However, hearing impairment has been linked to accelerated cognitive decline [6,8], and increased incidence of neurodegenerative pathologies, like Alzheimer’s disease (AD) [7,9,10]. Very recently, the Lancet consortium has established hearing loss as the major potentially modifiable risk factor for dementia [1], with significant implications for early treatment [7,9]. Indeed, while to date, there are no effective cures to counteract brain neurodegeneration, hearing impairments can be treated with prostheses or cochlear implants. Thus, understanding pathogenic mechanisms potentially linking hearing loss and dementia could have significant clinical implications, paving the way for the development of new therapeutic strategies. In this scenario, a possible candidate is oxidative damage with the consequent depletion of the endogenous antioxidant system. Indeed, oxidative stress is a well-known cause of hearing loss [11,12] and it is a common feature of several neurodegenerative disorders, such as AD [13]. Both cochlear cells and central nervous system (CNS) neurons are crucial targets of oxidative insult due to the high levels of energy required for their physiological functions, the abundant number of mitochondria, and their high consumption of oxygen [14,15]. Specifically, the hippocampus, the brain structure playing a critical role in memory [16], has been shown to be particularly susceptible to hearing loss-related oxidative stress [17]. In this review article, we will focus on redox imbalance as a common pathogenetic factor linking hearing loss and dementia. Understanding the pathological mechanisms responsible for these two conditions will aid in developing clinical interventions for the prevention and treatment of these diseases and their mutual interactions. We will start by discussing evidence demonstrating major cellular mechanisms of auditory processing and cognition. Afterwards, we will focus on sensorineural hearing loss characteristics and on the impact of this phenomenon on auditory and extra-auditory brain structures. Then, we will discuss the role of redox imbalance as a mechanism underlying both hearing loss and dementia. We will conclude by reporting data supporting the use of antioxidants as a therapeutic strategy for hearing loss and neurodegenerative diseases.

## 2. Auditory Processing and Cognition

**Figure 1:**
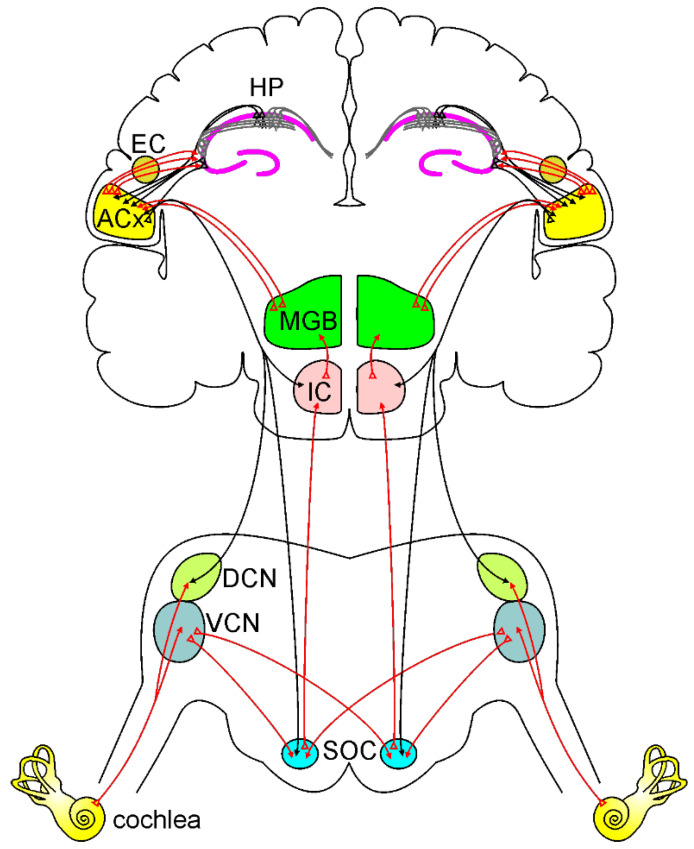
Ascending and descending auditory pathways.

Hearing ability is essential in everyday life, ensuring environment interaction, the identification of damaging stimuli, cognitive ability (i.e., auditory attention, auditory memory, language), and, not least, social interaction. The auditory system, which is highly complex anatomically and physiologically [18], allows for the identification, classification, and recognition of sounds. Sound waves coming from the surrounding environment are captured by the peripheral auditory structures, including the outer ear (pinna), eardrum (tympanic membrane), middle ear bones (malleus, incus, and stapes), and the cochlea [18]. The cochlea houses an arrangement of highly specialized cell types located in the organ of Corti and is responsible for sound mechanoelectrical transduction [18]. Specifically, there are two major classes of hearing sensory cells: the inner (IHCs) and the outer hair cells (OHCs). The IHCs, the hearing receptors, are arranged in one row on the basilar membrane in the organ of Corti, and they play a key role in transducing sound-evoked mechanical motion into electrical signals, leading to synaptic glutamate release at the terminals of the primary auditory nerve fibers, connecting sensory cells to spiral ganglion neurons (SGNs) [19]. The IHCs-mediated release of glutamate depolarizes the sensory cells starting auditory perception. The more numerous OHCs, arranged in three rows and named the “cochlear amplifier”, can contribute to IHC signal transduction by amplifying the motion of the basilar membrane to enhance sensitivity and frequency selectivity [20]. Electric stimuli leave the cochlea reaching central auditory nuclei via the auditory nerve. It transmits auditory information to auditory brainstem nuclei, including the cochlear nucleus, superior olivary complex, lateral lemniscus, and inferior colliculus, to finally reach the medial geniculate nuclei of the thalamus, before achieving the auditory cortex (ACx), (Figure 1) [21]. Of note, unlike what happens in other sensory systems, neurons within the auditory pathway have crossing fibers, suggesting that both ipsilateral and contralateral side information are crucial for auditory processing. Therefore, all levels of the central auditory system receive and process information from both the ipsilateral and contralateral sides [22]. Besides the ascending pathway from the cochlea to the ACx, there is also a descending pathway regulating auditory processing (Figure 1). Indeed, ACx has bilateral direct top-down projections to the inferior colliculus, superior olivary complex, and cochlear nuclei [23], so that connections between descending, ascending, and crossing fibers make the auditory system highly interconnected. Corticofugal circuits are also involved in several “auditory-cognitive tasks.” Such higher-order functions originate from many brain regions (i.e., prefrontal cortex, hippocampus, limbic circuits) that have either direct or indirect connections with ACx [24,25]. Auditory perception requires extremely complex central processing, and it has been considered an active cognitive task to understand and recognize environmental sounds [26,27]. Recently, several studies supported the hypothesis of a “cognitive ear” [7,28], underlying the strong relationship between auditory and cognitive functions. Indeed, auditory perception relays on several processes involving “auditory cognition” [27]. The ability to extract meaningful information from the complex everyday sound environment is fundamental in different listening conditions [29,30]. Listening to speech in background noise is an example of how auditory scene analysis is a very complex task: after “pre-cognitive” processing in the auditory brainstem [31], the auditory information, such as speech or voice features, is analyzed [32,33,34] extracting it from background noise to obtain hearing percepts called “auditory objects” [35,36]. Then, such auditory objects must be matched to stored auditory representations to be recognized [27]. It is evident that all these processes require high cognitive functions, such as attention, executive functions, working memory, and emotions. Finally, the link between hearing and cognition is also supported by clinical studies, showing that alterations in auditory processing are typical features of several psychiatric (i.e., schizophrenia, anxiety, depression) [37] and neurological (i.e., autism spectrum disorders, dementia) diseases [38,39].

A simplified model of the ascending auditory pathway is shown in red. The neural signal travels from the cochlea to the ventral and dorsal cochlear nuclei (VCN and DCN). Next, some of the axons cross the midline to the superior olivary complex (SOC), terminating in the inferior colliculus (IC) and to the medial geniculate body (MGB). Other fibers project ipsilaterally to SOC and IC, terminating MGB. Then information reaches the auditory cortex (ACx). Note that the ACx can send and receive information from the hippocampus (HP) directly or through the entorhinal cortex (EC). The descending auditory pathway is shown in black. Corticofugal projections from the ACx reach IC, SOC, and cochlear nuclei, modulating cochlear response.

## 3. Sensorineural Hearing Loss

Sensorineural hearing loss (SNHL) is the most common sensory disorder in humans: it has been estimated to affect about 360 million people worldwide, and more than half of the population over 60 years of age reports hearing impairment [40,41]. SNHL is characterized by hearing loss primarily due to the degeneration of the sensory-neural epithelium of the cochlea. The loss of hair cells resulting from damaging environmental factors exposure (i.e., noise, ototoxic drugs) or endogenous factors (i.e., aging, genetic susceptibility) can induce a permanent loss of sensitivity and frequency selectivity, leading to SNHL. Indeed, sensorineural cochlear tissue has very limited repair capacity, considering that the cochlear hair cells and neurons do not regenerate, making any cellular loss permanent. Thus, understanding the mechanisms underlying cochlear degeneration in SNHL and improving knowledge of cochlear pathophysiology is crucial for preventing hearing loss and for developing therapeutic strategies.

### 3.1. Noise-Induced Hearing Loss

Among the different exogenous damaging factors leading to hearing impairment, noise exposure is the most common cause of acquired SNHL in industrialized countries [42,43]. Of note, noise-induced hearing loss (NIHL) is also the most potentially preventable cause of auditory impairment. Indeed, the prevalence of NIHL, mainly related to exposure to recreational, environmental, and occupational noise, is about 16% of the adult population worldwide [44,45], accounting for approximately 11% of all occupational illnesses [46]. NIHL is usually characterized by the elevation in hearing thresholds [12,47,48], alteration of speech perception, and auditory processing disorders [49,50]. However, it could also be associated with a range of other auditory symptoms, such as phantom sound tinnitus, and hyperacusis, the increased sensitivity to loud sounds [51,52], as well as non-auditory symptoms, including sleep disorders, cognitive impairment, or social isolation due to reduced communication skills [44,53]. Cochlear damage induced by noise greatly depends on the noise characteristics, including the duration of the exposure, the frequency, the intensity, and the pattern of repetition [54]. Hearing loss associated with mild noise exposure (i.e., <100 dB sound pressure level, SPL) can lead to temporary threshold shift (TTS), probably due to reversible damage to the stereocilia of hair cells [55]. This cochlear impairment is reversible, and hearing can recover within a few weeks [41]. On the other hand, high-intensity (>120 dB SPL) or repeated/chronic noise exposures can cause irreversible hearing loss, leading to permanent threshold shift (PTS). Irreversible hearing loss is mainly due to cochlear hair cell injury, with permanent damage to their micromechanics properties and to the loss of SGNs [56]. Sensory damage to hair cells occurs very early, by minutes to days after exposure [57], whereas the damage to the neural compartment is slower, and the death of SGNs occurs by months to years after noise insult [58]. However, studies on animal models of NIHL have demonstrated that synaptic connections between hair cells and cochlear neurons were affected before the hair cells were damaged in the acute phase of noise exposure [59,60]. This can lead to a “hidden hearing loss”, resulting in IHC synapse damage (synaptopathy) due to an excess of Ca^2+^ influx and glutamate excitotoxicity [12,61]. Clinically, this condition is manifested by poor speech recognition ability, often observed in patients exposed to noise [49].

Susceptibility to NIHL differs among individuals due to a combination of genetic and environmental factors. It has been estimated that the heritability of NIHL is ~36% [62]. However, single nucleotide polymorphisms (SNPs) and genome-wide association studies (GWAS) have identified a small number of potential susceptibility genes, mainly involved in pathways regulating oxidative stress response and K^+^ channels expression and activity [63,64,65]. Finally, NIHL became a significant public health problem even for the interaction with accelerated age-related hearing loss (ARHL) [47,66], given the concomitant high prevalence of noise exposure and aging in our society. Indeed, recent epidemiological and experimental studies highlighted the consequences of hearing deprivation in the elderly, including poor quality of life, depression, accelerated cognitive decline, increased risk of dementia, hospitalizations, and early mortality [1,5,17,28,67].

### 3.2. Age-Induced Hearing Loss

ARHL, or presbycusis, refers to the age-dependent decline in hearing sensitivity, leading to an increased auditory threshold in aged subjects. In the United States, more than 50% of people over 70 years show ARHL [68]. Moreover, the prevalence of ARHL is expected to increase substantially in the worldwide population due to an increase in the aging population, environmental risk factors (i.e., noise pollution), and wrong lifestyles [69]. Presbycusis is considered a multifactorial disease, usually characterized by progressive, bilateral, and symmetrical hearing loss, initially involving the high-frequency region of the hearing spectrum and progressing later in the low-frequency range [41,70]. The age-dependent reduction in threshold sensitivity is generally associated with altered speech discrimination, as well as sound detection and localization, particularly in a noisy background. These limitations in communication skills can often lead to a high risk of social isolation, depression, and dementia [71]. In clinical practice, we can distinguish three major forms of ARHL, identified and classified by Schuknecht based on both the audiometric profile and the localization of cochlear damage: (1) “sensory presbycusis,” associated with a hearing loss in the high-frequency range and due to hair cell loss starting from the basal cochlear region; (2) “neural presbycusis,” characterized by more severe impairment in speech discrimination for pure tone threshold, due to prominent damage of both cochlear neurons and primary afferent fibers; and (3) “strial” or “metabolic” presbycusis, characterized by hearing loss across all frequency ranges, due to the degeneration of the stria vascularis [72]. The mechanisms underlying why and how aging processes target different cochlear structures remain elusive [73], mainly because of the complexity of etiological factors for presbycusis, including intrinsic (i.e., genetic predisposition, epigenetic factors, aging), and extrinsic factors (i.e., noise exposure, ototoxicity, smoking) contributing to ARHL phenotypes.

Studies in presbycusis patients have estimated that from 25% to 75% of the variance in this pathology has a genetic component [74]. Research on animal models identified some candidate genes for presbycusis, including ARHL gene 1 (*Ahl1*), localized in chromosome 10, *Ahl2* [75] on chromosome 5, and *Ahl3* on chromosome 17 [76]. Thus, mouse strains harboring the *Ahl* allele, such as the C57BL/6 mouse model, are considered valuable models of ARHL [70,77]. Variants of the gap junction protein beta-2 (*Gjb2*) and gap junction protein beta-6 (*Gjb6*) genes, encoding connexin 26 (Cx26) and connexin 30 (Cx30), respectively, have been linked to the deafness DFNB1, the most frequent non-syndromic hearing loss in the Mediterranean population [78]. Recent studies have suggested that alterations in inner ear connexin expression and function contribute to the etiopathogenesis of ARHL. Indeed, it has been shown that homozygous offspring with targeted deletion of Cx26 in the epithelial gap junction network of the cochlea (Gjb2−/− mice) failed to acquire hearing function [79], whereas the heterozygous (Gjb2+/−) mice show an accelerated presbycusis phenotype that appears at 6 months of age [80]. Moreover, we found that in Cx30 knock-out (KO) mice (Cx30 ΔΔ mice), ARHL and cochlear dysfunctions were exacerbated, and hearing thresholds were significantly higher at 12 months of age in KO mice with respect to age-matched wild-type animals [81]. Namely, these data show that both *Gjb2* and *Gjb6* mutations are candidate genetic risk factors for ARHL.

Increasing evidence from both clinical and animal studies indicates that events and injuries occurring in life may contribute to the vulnerability of inner ear structures to aging processes [41,70,82]. Among these, NIHL has been considered a risk factor for ARHL [83]. Indeed, both independently or synergistically, noise exposure and aging have long been associated with the development of hearing loss in the adult/elderly population [43,61,84]. Several studies suggested that NIHL during aging causes acceleration and/or worsening of ARHL [70,73,82,83]. Mouse strains exhibiting ARHL, such as the C57BL/6 mice, are more vulnerable to noise insult compared with mouse strains on which ARHL has not been observed [85]. Similarly, we recently demonstrated that in C57BL/6 mice, acoustic trauma exposure at a young age (2 months of age) accelerates the onset of ARHL [86], indicating that there is a long-term effect of early (juvenile period) noise exposure on ARHL. Moreover, in the same mouse model, we found that hearing loss, along with accelerated presbycusis onset, induced persistent synaptic alterations in the ACx. This was associated with decreased memory performance and oxidative-inflammatory injury in the hippocampus [87].

## 4. Impact of Sensorineural Hearing Loss on Brain Auditory and Extra-Auditory Structures

It has been reported that hearing impairment causes alterations in both auditory and “extra-auditory” brain regions involved in the non-lemniscal path, such as the hippocampus [17,88]. Indeed, auditory sensory deprivation induced by cochlear damage can trigger up-spread damage leading to maladaptive plasticity [89,90] and changes in excitatory, inhibitory, and neuromodulator networks along the central auditory pathway [91,92,93]. We previously demonstrated that NIHL led to structural remodeling in pyramidal neurons of layer II/III of the ACx, causing decreased dendritic spine density and altering dendritic complexity [89,90,94]. It is also known that hippocampal neurons can process sensory inputs, including acoustic stimuli [95,96,97,98] to create spatial memories [99,100,101]. Indeed, lesions of the perforant path, which relay auditory information from the entorhinal cortex to the hippocampus, disrupt auditory-evoked neuronal responses in the hippocampus [97]. Additionally, a direct projection from CA1 to the ACx has been found [102]. Auditory potentials evoke neuronal responses in the hippocampus [103] and hearing loss impacts hippocampal functions altering neurotransmitter levels [104,105], increasing Aβ and hyperphosphorylated tau levels [106,107,108], decreasing neurogenesis [109,110,111] and increasing oxidative stress [112]. Moreover, it has been recently demonstrated that hearing loss induced by ototoxic drug administration can increase inflammatory cytokine levels in the hippocampus, causing neuronal death together with an up-regulation of dementia-related protein expression [113]. In line with this evidence, we recently demonstrated that NIHL in a pre-symptomatic phase (at 2 months of age) accelerates cognitive decline in a mouse model of AD (3×Tg-AD mice), anticipating memory dysfunctions with respect to the expected time course of the pathology and causing long-lasting functional, morphological, and molecular neuronal alterations not only in the ACx but also in the hippocampus [67]. Furthermore, increasing experimental evidence from animal models of presbycusis, including ours, highlights that noise exposure exacerbates aging mechanisms, making cochlear structures more susceptible to aging processes [41,70,82,86]. Recently, we evaluated the effect of repeated noise exposures early in life on age-related cochlear dysfunctions in C57BL/6 mice. As mentioned above, these animals show early onset of ARHL, with increased auditory thresholds spreading from high to low-frequency regions with advancing age [70,114]. We found that noise exposure can anticipate the presbycusis phenotype, exacerbating cochlear damage induced by oxidative stress, inflammation, and vascular dysfunction [86].

## 5. Redox Imbalance as an Underlying Mechanism in Hearing Loss and Dementia

Oxidative stress refers to the failure of the redox balance between the production of oxidants and the antioxidant defense system activity [115]. It has been associated with a wide range of diseases, including cardiovascular disorders, muscle dysfunction, allergy, and cancers, but also sensory and cognitive neurodegenerative diseases [13,116,117]. Reactive oxygen species (ROS) are oxygen-based molecules comprising free radical superoxide (O_2_•−), hydroxyl radical (•HO), and singlet oxygen (^1^O_2_), as well as non-radical species such as hydrogen peroxide (H_2_O_2_) formed by the partial reduction of oxygen [118,119]. The principal source of ROS is the mitochondrial respiratory chain, which produces oxidants as part of aerobic respiration [115]. ROS can directly oxidize macromolecules, including membrane lipids, structural proteins, enzymes, and nucleic acids, triggering stress signals that induce apoptosis, stress resistance, or senescence, consequently leading to aberrant cell function, and death [120]. Indeed, ROS initiates the mitochondria-mediated apoptotic cascade by cytochrome-c release, the activation of the initiator caspase-9, and its effector caspase-3. A primary target for ROS is the plasma membrane, where ROS can induce lipid peroxidation, as shown by the accumulation of toxic lipid peroxides, malondialdehyde, and aldehydes, including peroxynitrite and 4-hydroxynonenal [12,121]. Moreover, ROS can induce oxidative modifications of the DNA, leading to the degradation of bases, mutations, translocations, and abnormal cross-linking with proteins [122,123]. Indeed, mitochondria maintain their own DNA called mitochondrial DNA (mtDNA), which is critical to the proper function of mitochondria [124]. mtDNA is prone to mutation induced by oxidative damage due to its proximity to the site of ROS generation and to the relative lack of DNA-protective histones and efficient repair mechanisms [125]. Thus, ROS production is responsible in part for producing mtDNA damage by causing mutations/deletions in the mitochondrial genome [126]. ROS-induced mtDNA damage can lead to the synthesis of functionally impaired respiratory chain subunits, causing respiratory chain dysfunction [122].

In physiological conditions, maintaining a redox balance ensures an adequate intracellular ROS level, which is essential to regulate signaling pathways [127,128] and cellular homeostasis [128]. However, when ROS concentrations increase, exceeding the capacity of the endogenous antioxidant to maintain a redox balance, they become toxic [14,128], leading to oxidative stress status. The endogenous antioxidant system involves enzymes, such as superoxide dismutase (SOD), catalase (CAT), glutathione peroxidase (GPx), glutathione reductase (GR), and other non-enzymatic compounds, such as glutathione (GSH), thioredoxin (Trx), vitamins A, E, and C, flavonoids, trace elements, and proteins (i.e., albumin, ceruloplasmin, and metallothionein), that has been shown to reduce different oxidants [129].

Because of their high oxygen consumption, high lipid content, abundant mitochondria, and high energy requirement, neurons and hair cells are particularly vulnerable to damage caused by ROS. Oxidative stress has been proposed to be the pathogenic core of both SNHL [11] and neurodegenerative disease [130]. Therefore, all these considerations have led to the idea that oxidative stress could be considered a common pathogenic factor shared between hearing loss and cognitive decline.

### 5.1. Role of Oxidative Stress in Sensorineural Hearing Loss

**Figure 2:**
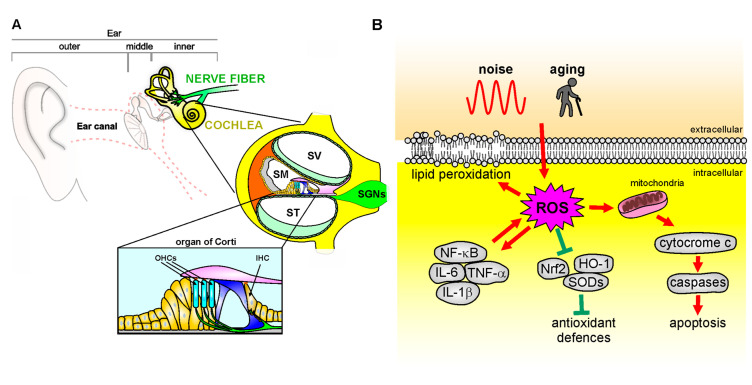
Mechanisms of oxidative stress in the cochlea. (**A**): Schematic representation of cochlear anatomy, with the organ of Corti, containing one row of inner hair cells (IHC) and three rows of outer hair cells (OHC), the main structure involved in oxidative stress attack. SV: Scala Vestibuli; SM: Scala Media; ST: Scala Tympani; SGNs: Spiral Ganglion neurons. (**B**): Noise or aging insults can induce oxidative damage leading to cytochrome c release from the mitochondria, which activates the caspase apoptotic pathway. ROS overload causes in parallel an imbalance of endogenous antioxidant enzymes [Nrf-2, heme oxygenase-1 (HO-1), superoxide dismutases (SODs)] and an increase of pro-inflammatory markers (NF-κB, TNF-α, IL-1β) that contributes to pro-apoptotic pathway activation, leading to cell death. Finally, ROS leads to increased lipid peroxidation, affecting plasma membrane fluidity.

Cochlear mechanoelectrical transduction is a complex mechanism that requires high metabolic demands to maintain large electrochemical gradients, which is essential for mechanoelectrical transduction. The high metabolic demand, in turn, renders the cochlear structures particularly vulnerable to oxidative stress damage. Indeed, it has been demonstrated that alteration in redox balance leads to oxidants accumulation, damaging several elements such as sensory cells, stria vascularis, and afferent neurons, causing cell death and hearing loss [11,89,131,132]. ROS accumulation promoted an increase in lipid peroxidation markers (i.e, 4-HNE or isoprostanes), contributing to cell damage [121,133,134]. Among the enzymes producing ROS, the NADPH oxidase family (NOX) has been proven to catalyze the electron transport from NADPH to oxygen molecules to accelerate ROS production [135]. Transgenic mice expressing the human NOX4 isoform were more susceptible to noise insult, although no alterations in basal hearing threshold were observed [136]. We found that noise-mediated oxidative stress modulates, through lipid peroxidation, the plasma membrane fluidity of OHCs as a consequence of NAD(P)H expression [121]. Lipid peroxidation affects the physiological integrity of plasma membrane and lipid-protein interactions, altering membrane fluidity [137]. The fluidity of the plasma membrane is critical to OHC function, ensuring lateral wall micromechanics [138]. After noise exposure, we found changes in OHC redox state as a consequence of an increased NAD(P)H oxidation [139,140], followed by a rise of lipid peroxidation, correlated with a decrease in membrane fluidity, suggesting a cause–effect relationship [121]. Interestingly, we also studied the kinetics of damage onset, showing that reduced NAD(P)H was characterized by a fast oxidation time (~0.36 h). This, in turn, affects plasma membrane organization (starting from ~7.6 h), triggered by lipid peroxidation (occurring within ~4 h). Thus, lipid peroxidation induced by oxidative stress, with the consequent decrease of plasma membrane fluidity, leads to a drop of NAD(P)H, reaching lower levels in ~9.4 h [121]. These findings are of great translational interest, indicating that there is a critical post-traumatic period within an antioxidant intervention that could be more effective in preventing OHC oxidative damage. Experimental evidence also links redox imbalance to ARHL [141,142]. Indeed, mice lacking SOD1 showed morphological alterations in the organ of Corti, SGNs, and stria vascularis, associated with presbycusis phenotypes [143]. Moreover, an early increase of 7,8-dihydro-8-oxoguanine (8-oxoG), a key biomarker of mitochondrial and nuclear DNA damage induced by oxidative stress [144], was observed in the cochleae of SAMP8 mice, a mouse model of cochlear senescence [142]. The role of mitochondrial damage in ARHL was also confirmed by studies reporting increased mitochondrial DNA deletions and mutations in patients with ARHL, compared to normal-hearing subjects [145], as well as in animal studies demonstrating direct evidence for mitochondrial dysfunctions in ARHL [146,147,148]. Major mtDNA mutations involve genes encoding mitochondrial oxidative phosphorylation complexes, leading to an impairment of its activity [149]. Mutations altering mtDNA genomic stability, such as defects in the DNA polymerase γ (POLG), that maintains mtDNA replication fidelity [150,151], or the OPA1 gene [152,153], which is involved in mitochondrial fission, leading to premature hearing loss. Moreover, mtDNA mutations affect cochlear function by leading not only to mitochondrial dysfunction but also metabolic energy alterations and triggering apoptotic processes [147]. We also reported that mice lacking p66(shc), a proapoptotic protein involved in mitochondrial ROS production [154,155] showed a delayed age-dependent decline of hearing function [156]. At the same time, these mice were resistant to oxidative stress, inflammation, and vascular dysfunction induced by noise exposure with respect to wild-type animals [156]. These data confirmed the crucial role of oxidative stress and redox signaling in both ARHL and NIHL, indicating that genetic predispositions and environmental risk factors can interact in determining noise and age-induced hearing impairment. The crucial role of oxidative stress as a common mechanism shared between NIHL and ARHL (Figure 2), has been documented by several studies, including ours [12,41,157]. Moreover, we recently demonstrated that noise exposure in 2-month-old C57BL/6 mice exacerbates ARHL, usually occurring at about 9 months of age in these animals, causing a hearing impairment spanning all frequencies at 6 months of age, exacerbating cochlear senescence through oxidative stress, inflammation, and vascular dysfunction mechanisms [86]. ROS generation can also lead to the production of pro-inflammatory cytokines (Figure 2) that can further worsen cochlear damage [158,159]. Several studies have demonstrated that ROS interacts with the nuclear factor kappa-B (NF-κB), a key transcription factor in the inflammatory signaling pathway [117,160]. NF-κB-dependent genes have been shown to modulate ROS levels and, in turn, NF-κB activity is also affected by the free radical amount [12,161,162]. Moreover, noise and aging have been shown to trigger cochlear production of cytokines [163], such as interleukin 1-β (IL-1β) of TNF-α [159]. Recently, we demonstrated that acute noise exposure triggers crosstalk between oxidative stress and inflammation [164]. Our results showed that ROS production after noise exposure leads to increased inflammatory markers, probably through the downregulation of PPARs, which are ligand-regulated transcription factors involved in oxidative stress and inflammatory pathways [165], regulating cellular oxidative balance [166]. Indeed, noise exposure induced a down-regulation of PPAR cochlear expression, together with an increase of both oxidative and inflammatory markers. By decreasing oxidative stress with antioxidant treatment (Q-ter), PPAR expression returned to control values, reactivating the negative control on inflammation and, in turn, decreasing oxidative stress, in a feedback loop [164]. Thus, oxidative stress can be considered the first damaging factor in cochlear insult, triggering, consequently, inflammatory processes that, in turn, exacerbate oxidative damage, in a vicious cycle [164].

### 5.2. Role of Oxidative Stress in Neurodegenerative Diseases

**Figure 3:**
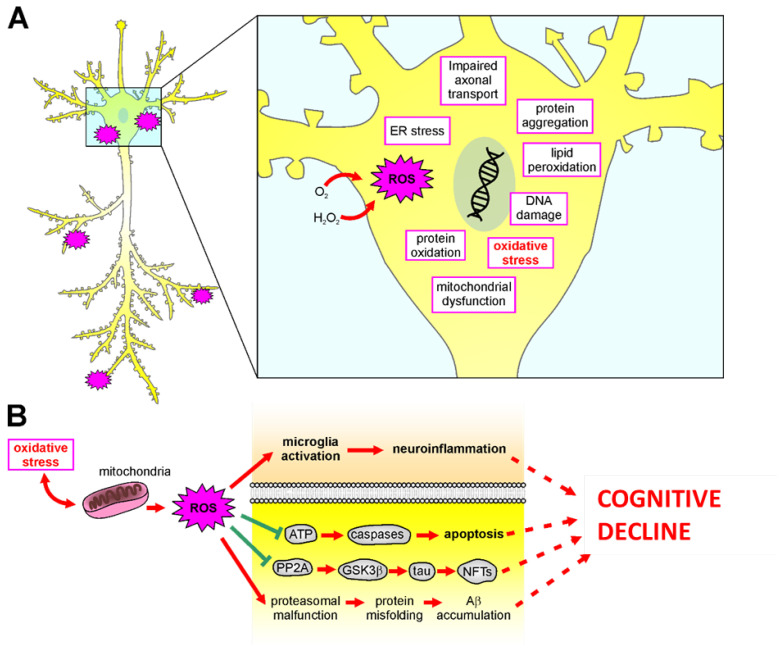
Mechanisms of oxidative stress in the brain. (**A**): Schematic representation of all damaging molecular mechanisms involved in ROS-mediated insult in neurodegenerative diseases. (**B**): Oxidative stress causes mitochondrial damage and ROS release, damaging neurons and leading to neuronal dysfunctions and cognitive decline by: (1) inducing microglia activation leading to neuroinflammation; (2) impairing ATP release, leading to caspase-dependent apoptotic pathways; (3) increasing neurofibrillary tangles (NFTs) accumulation caused by hyperphosphorylation of tau thought decreased PP2A activity and increased GSK3β; (4) facilitating misfolding protein aggregation as Ab accumulation.

Neurodegenerative diseases refer to several uncurable and debilitating pathological conditions, including AD, Parkinson’s disease (PD), Huntington’s disease (HD), amyotrophic lateral sclerosis (ALS), and others, characterized by progressive damage in neural death, responsible for alterations in motor or cognitive functions [167]. The pathogenesis of these diseases remains largely unknown; however, several pieces of evidence suggest a crucial role of oxidative stress [13]. In this review, we will focus on a discussion of the role of oxidative stress in AD (Figure 3), although ROS impairments have also been observed in PD, HD, and ALS. AD is the most prevalent disorder leading to dementia worldwide [168]. Clinically, AD patients show progressive brain atrophy, usually starting from medio-temporal brain regions, resulting in memory impairment and executive dysfunction, also accompanied by neuropsychiatric symptoms [169]. The extracellular deposition of amyloid beta (Aβ) plaques and the accumulation of intracellular tau neurofibrillary tangles (NFT) are considered the main pathological hallmark of AD. Additionally, at early stages, oligomers of these misfolded proteins are known to cause several neuronal dysfunctions affecting synaptic signaling and mitochondrial functions [170,171,172], favoring ROS production and oxidative stress [172]. Aβ oligomers deplete Ca^2+^ storage in the endoplasmic reticulum, resulting in an exciting cytosolic Ca^2+^ load. In response to cytosolic Ca^2+^ increase, endogenous GSH levels are reduced, and the cellular free radical amount rises [173]. Similarly, Aβ oligomers insertion into the bilayer of the plasma membrane can cause ROS accumulation, initiating lipid peroxidation of membranes, followed by intracellular protein and nucleic acid oxidation [174]. In turn, free radical overproduction can play a crucial role in Aβ accumulation (Figure 3) [175,176]. Aβ has been shown to play an important role in activating the apoptotic pathway [177] by increasing the activity of calcineurin, which in turn triggers Bcl-2-associated death promoter, leading to cytochrome c release from mitochondria and caspase activation [178]. Accordingly, reduced cytochrome oxidase activity and mitochondrial defect have been found in AD patient tissues [179]. The role of mitochondrial dysfunctions in AD has also been confirmed by several data demonstrating a reduced complex IV activity of the respiratory chain in hippocampal mitochondria of AD patients and in AD cybrid cells [106,180]. Moreover, an early decrease in mitochondrial complex activity and abnormal expression of mitochondrial fission proteins has also been found in AD brains [181]. As described for sensorineural hearing loss, mtDNA mutation can play a role also in the pathogenesis of neurodegenerative diseases. Indeed, the crucial role of mtDNA in proper cognitive function is supported by studies demonstrating increased oxidation of mtDNA in AD brains [126,182]. Additionally, patients affected by primary mtDNA mutations show cognitive deficits, similarly to what observed in subjects with dementia [183]. Moreover, levels of oxidized nucleic acids in mtDNA were found to be significantly increased in early phase of AD and MCI [184], suggesting that this can be considered an early hallmark of pathology. Mitochondria are also richly expressed at the synaptic level, due to the local elevated energy requirement necessary to sustain all steps of chemical synaptic transmission, including Ca^2+^ modulation [185]. It has been shown that Aβ protein accumulation at the levels of the synaptic mitochondria [186] affects synaptic function [106], probably interacting with mitochondrial matrix proteins [187]. Aβ-induced ROS overproduction can also lead to tau hyperphosphorylation through the activation of p38 mitogen-activated protein kinase (MAPK). Increases in tau phosphorylation have also been shown to be directly mediated by ROS in vitro [109,188]; in addition, lipid peroxidation facilitates the aggregation and hyperphosphorylation of tau [189]. Furthermore, ROS causes inhibition of phosphatase 2A (PP2A) [190], which facilitates glycogen synthase kinase (GSK) 3β activation, involved in tau phosphorylation (Figure 3) [191]. Finally, as described above for hearing impairment, an interplay between oxidative stress and inflammation also plays a crucial role in AD pathogenesis. Indeed, inflammation has been shown to increase cytokine expression and free radicals, contributing to AD progression [192,193]. Neuroinflammation and glial response can exacerbate Aβ accumulation through microglia activation [194], which plays a key role also in enhancing oxidative stress levels, with a feedback loop exacerbating oxidative-inflammatory damage and leading to synaptic dysfunctions and neuronal damage (Figure 3) [195]. Indeed, increased levels of the pro-inflammatory cytokine TNF-α and decreased levels of the anti-inflammatory cytokine TNF-β have been found in patients with mild cognitive impairment who progressed to AD [196]. Moreover, Aβ accumulation leads to alterations in microglia and astrocytic metabolism [197,198], and triggers the release of neuroinflammatory mediators, promoting, in turn, synaptic dysfunctions and neurodegeneration [199,200].

### 5.3. Oxidative Stress as a Common Pathogenetic Cause

Among the potential theories proposed to explain the link between sensorineural hearing loss and dementia, the existence of a possible common pathogenetic mechanism affecting the auditory periphearl organ (the cochlea), causing hearing loss, and barin structures (hippocampus), causing cognitive decline, has been considered [201]. As discussed above, one common pathogenetic cause can be the increase of oxidative stress and redox imbalance spreading from the cochlea to the brain. Indeed, there is a body of literature suggesting that peripheral hearing loss and hippocampal redox changes may share common mechanisms which are highly sensitive to metabolic insults [202,203]. However, how can sensorineural hearing loss can be linked to hippocampal dysfunction via oxidative stress? Although several studies reported an increase of oxidative stress markers in the hippocampus following hearing loss [67,112], the mechanism by which hearing loss induces this alteration in the hippocampal redox state remains elusive.

It has to be considered that hearing loss induced by noise or aging causes an up-spread deafferentation in the auditory system [89,90], so that it is reasionable that hearing loss impacts neuronal activity in the auditory pathway [204], leading to alteration in spontaneous and driven spiking properties in neuronal structures involved in both lemniscal and non-lemniscal path, such as the hippocampus. Specifically, the lemniscal pathway involves projections from the auditory cortex to the entorhinal cortex that then projects to the hippocampus [103,205,206,207]. Additional pathways involving projections from the cochlear nucleus to the hippocampus have also been proposed [103,208,209] and also a direct projection from CA1 hippocampal region to the ACx has been reported [102] (Figure 1). In line with these considerations, it has been shown that NIHL can induce neurotransmission alterations in extra-auditory CNS structures [210], leading to an increase of hippocampal glutamate content [211,212]. Specifically, acute or subacute exposures to noise have been associated respectively with an increased [213] or decreased [214] expression of the NR2B subunit of NMDA receptor in the hippocampus, suggesting that this receptor may be modulated over time after excessive exposure to glutamate. Similarly, increased hippocampal NR2B expression, along with memory impairment have been reported in an animal model of ARHL [105]. Of note, the increase of glutamate content can induce excitotoxicity, mainly through excessive stimulation of NMDA receptors, and excessive glutamate signaling can lead to a shift in neuronal redox potential [213]. Moreover, oxidative stress and redox unbalance can lead to an accumulation of extracellular glutamate [214]. Thus, excitotoxicity and ROS generation are related in a positive feedback loop. Indeed, excitotoxicity is associated with the impairment of calcium buffering and increased generation of ROS [215]. On the other hand, excessive ROS production leads to glutamate receptor overstimulation that can, in turn, transform glutamatergic neurotransmission in a mediator of intracellular oxidative stress [210]. Finally, several studies also demonstrated that hearing loss induced by noise or ototoxic drugs can affect hippocampal neurogenesis [109,113,216,217]. Neurogenesis is crucial for learning and memory processes [218,219] and it is vulnerable to oxidative stress [220], as well as to excitotoxicity damage [221]. In line with these considerations, one possible hypothesis is that diminishing cochlear inputs due to hearing loss, as a result of oxidative stress damage and alteration in glutamatergic activity, affects the CNS through the same mechanisms, leading to hippocampal dysfunctions, considering that this structure is involved in the extra-auditory pathway and it is particularly prone to oxidative stress damage [203,222].

Of course, a deeper understanding of the molecular mechanism relating to hearing loss and hippocampal dysfunction is needed, and further works on this topic are necessary to shed light on the role of oxidative stress as a common pathological marker linking hearing loss and dementia.

## 6. Use of Antioxidants as Targeted Therapeutics

Considering the prominent causal role of oxidative stress in the pathogenesis of hearing loss and dementia, several studies addressed the possibility to target redox unbalance in cochlear or brain structures to counteract oxidative stress [12,223,224,225], as summarized in Table 1. For this reason, the use of exogenous antioxidants with scavenger properties and with the ability to potentiate the endogenous antioxidant system has been widely tested in several animal models, with promising results. However, as discussed below, some limitations arise from the low bioavailability of antioxidant molecules and controversial results have been obtained when translating antioxidant therapy from animal studies to clinical trials.

Among all molecules with antioxidant activity, N-acetyl, L-cysteine (NAC) is probably the most studied against noise-induced oxidative stress. Indeed, NAC has been tested in several experimental conditions, animal models, and dosages [226,227,228]. NAC can directly scavenge H_2_O_2_, and hydrogen radicals and it is considered a major contributor to the maintenance of cellular GSH, acting as a substrate for its synthesis [228]. Combined treatment with more than one antioxidant molecule has also been demonstrated to be effective. For example, treatment of NAC and hydroxylated alpha-phenyl-tert-butylnitrone (4-OHPBN) decreased noise-induced hearing impairment and OHC loss [229]. Interestingly, the protective effect of using NAC in attenuating ARHL in SAMP8 mice, in conjunction with its ability to prevent age-induced memory impairment, has been demonstrated [230]. The effectiveness of NAC treatment has also been documented in neurodegenerative disease [231].

Vitamin E is considered one of the most important antioxidants in the brain, especially the α-tocopherol form [232]. The protective effect of α-tocopherol treatment has been demonstrated in animal models of hearing loss caused by ototoxic drugs [233,234] and by noise exposure [235]. Interestingly, α-tocopherol has also been considered a valuable antioxidant candidate against AD [236]. Indeed, epidemiological studies showed an association between supplementation with Vitamin E and decreased risk of developing AD [237,238], and increased α-tocopherol levels have been associated with lower levels of AD pathological features [239]. On the other hand, lower levels of Vitamin E have been found in AD brain patients [240,241,242].

Among direct scavenger modules, we studied the antioxidant ability of Coenzyme Q10 (CoQ10), as well as its analog Idebenone and its soluble form Q-ter, against cochlear oxidative insult [193,243,244,245]. Our results showed the potent ability of these antioxidants to attenuate hearing loss in different models of noise damage (acute or chronic noise exposures) [164,227,245]. Similarly, CoQ10 administration attenuates Aβ accumulation and reduces tau phosphorylation, mitigating AD phenotypes [246,247,248]. Moreover, CoQ10 levels significantly correlated with Aβ accumulation in dementia patients [249], and they were associated with a high risk of developing dementia [250]. Many studies also focused on the efficacy of natural compounds, able not only to scavenge oxidants but also to potentiate endogenous antioxidant systems [12,251]. Specifically, phenolic compounds, the natural antioxidants contained in most foods and beverages, can activate antioxidant genes [252,253]. The nuclear erythroid-2-like factor-2 (Nrf2) is a master cell homeostasis regulator that responds to dietary antioxidants, regulating the expression of several phase II cytoprotective genes. Under basal conditions, Nrf2 is sequestered by Keap1 in the cytoplasm, where it is targeted for ubiquitin-mediated proteolysis, whereas once activated by oxidative stress, Nrf2 translocates to the nucleus, where it binds the endogenous antioxidant response elements (ARE) in the DNA sequence, leading to the translation of endogenous antioxidant enzymes, such as heme oxygenase-1 (HO-1), nicotinamide adenine dinucleotide phosphate (NAD(P)H), SODs, enzymes involved in GSH metabolism and others [254,255,256,257]. Against SNHL, we tested the efficacy of several phenolic compounds, such as Curcumin [258], Ferulic acid [245,259], Rosmarinic acid [260,261] and Caffeic acid [262], showing that the administration of these natural products was effective in counteracting cochlear damage. In particular, we found a direct scavenger effect against oxidative stress, but also the ability of these natural compounds to indirectly potentiate endogenous antioxidant responses, favoring Nrf2 nuclear translocation in both hair cells and SGNs and, consequently, increasing expression of antioxidant enzymes, such as HO-1, GSH, and SODs [259,262]. Curcumin showed otoprotective properties by potentiating HO-1 expression in a model of hearing loss induced by an ototoxic drug [263], but also neuroprotective properties, by inhibiting the aggregation of various amyloidogenic proteins, such as Aβ and α-synuclein [264,265]. Rosmarinic acid showed protective properties in attenuating cochlear damage after acute acoustic trauma, by targeting the Nrf2-HO-1 assessment [260] and it is also shown to be effective in attenuating neurodegenerative processes, counteracting AD pathology [266,267,268,269,270] and improving cognitive performance in healthy young and aged subjects [271,272]. Resveratrol exhibits both oto- and neuroprotective activity, mainly by activating the endogenous antioxidant enzymes through the modulation of intracellular factors associated with oxidative stress (i.e., HO-1), neuronal energy homeostasis (i.e., AMP kinase), program cell death (i.e., Apoptosis induced factor—AIF) and longevity (i.e., sirtuins) [273,274]. Namely, experimental studies showed a protective effect of Resveratrol in attenuating cognitive dysfunctions [275] as well as both ARHL [276,277] and NIHL [278,279]. Notwithstanding the promising results obtained in experimental models, the use of antioxidants in clinical practice is still limited because of inconsistent and conflicting results obtained in clinical trials [266,280]. Moreover, it must be considered that some antioxidant molecules show hermetic properties, with both antioxidant and pro-oxidant effects, depending on the dosage used [259,281,282]. This has been called the “antioxidant paradox” [283], and it represents an important limitation of antioxidant therapy. Another crucial point is the poor bioavailability of several antioxidants, depending on numerous complex factors, such as the intake of competing nutrients, intestinal differences in absorption, age, gender, smoking, obesity, and genetic polymorphisms [284]. To overcome these defects, innovative strategies to improve drug delivery are ongoing, focused on the possibility of developing cyclodextrins and nanoparticles as drug carriers, which can be applied for enhancement of the solubility, stability, and absorption rate of antioxidants [285,286,287,288,289], with promising results.

**Table 1:** Antioxidant molecules showing efficacy in counteracting both hearing loss and Alzheimer’s disease and their molecular mechanisms (↑ increase; ↓ decrease).

Antioxidant Compound	Molecular Mechanisms	References
CoQ10, Q-ter (soluble form) and Idebeneone (Q-ter analogue)	↓ ROS ↓ antiapoptosis ↓ Aβ, ↓ pTau ↓ NF-kB ↓ pro-inflammatory cytokines ↑ SOD	[164,227,245,246,247,248,249]
Vitamin E and α-tocopherol	↑ GSH ↑ SOD	[233,234,235,236]
N-acetyl, L-cysteine (NAC)	↓ ROS ↑ GSH synthesis ↓ NF-kB	[226,227,228,230,231]
Caffeic acid	↓ ROS and RNS ↓ NF-kB and IL-1b ↑ Nrf2/HO-1 pathway ↓ pTau ↓ Aβ,	[261,290]
Curcumin	↑ Nrf2 signaling ↓ NF-kB ↓ Aβ, ↓ α-synuclein	[258,263,264,265].
Ferulic acid	↑ Nrf2/HO-1 pathway ↓ vascular damage ↓ Aβ	[245,259,291]
Rosmarinic acid	↑ Nrf2/HO-1 pathway ↓ NF-kB ↓ Tau and Aβ aggregation	[260,261,270]
Resveratrol	↑ PI3K/Akt pathway ↑ Nrf2 nuclear translocation ↓ NF-kB and MAPK pathways	[273,274,276,277,278,279]

## 7. Conclusions

Collectively, clinical and experimental evidence suggests a strong link between hearing and cognition, so hearing loss has been considered one of the main modifiable risk factors for cognitive decline. In this review, we discussed data showing that oxidative stress similarly impinges on inner ear and brain structures involved in cognitive functions by common pathogenic mechanisms. Thus, redox imbalance represents a key determinant of the vicious cycle between hearing loss and cognitive impairment both during “physiological” aging and in neurodegenerative diseases. A greater understanding of the molecular determinants and intracellular pathways linking hearing loss and dementia can have a crucial impact on treatment and prevention of these oxidative stress-dependent neural dysfunctions. Antioxidant therapy to counteract both hearing loss and cognitive impairment is a promising approach, also given the possibility of using dietary antioxidants such as polyphenols. However, further research is needed to understand in greater details the mechanisms linking cochlear and brain vulnerability to oxidative stress and to overcome antioxidant treatment limits, including low bioavailability.
